# Regulation of Small RNA Accumulation in the Maize Shoot Apex

**DOI:** 10.1371/journal.pgen.1000320

**Published:** 2009-01-02

**Authors:** Fabio T. S. Nogueira, Daniel H. Chitwood, Shahinez Madi, Kazuhiro Ohtsu, Patrick S. Schnable, Michael J. Scanlon, Marja C. P. Timmermans

**Affiliations:** 1 Cold Spring Harbor Laboratory, Cold Spring Harbor, New York, United States of America; 2 Watson School of Biological Sciences, Cold Spring Harbor, New York, United States of America; 3 Center for Plant Genomics, Roy J. Carver Co-Laboratory, Iowa State University, Ames, Iowa, United States of America; 4 Department of Plant Biology, Cornell University, Ithaca, New York, United States of America; The University of North Carolina at Chapel Hill, United States of America

## Abstract

MicroRNAs (miRNAs) and *trans*-acting siRNAs (ta-siRNAs) are essential to the establishment of adaxial–abaxial (dorsoventral) leaf polarity. *Tas3*-derived ta-siRNAs define the adaxial side of the leaf by restricting the expression domain of miRNA miR166, which in turn demarcates the abaxial side of leaves by restricting the expression of adaxial determinants. To investigate the regulatory mechanisms that allow for the precise spatiotemporal accumulation of these polarizing small RNAs, we used laser-microdissection coupled to RT-PCR to determine the expression profiles of their precursor transcripts within the maize shoot apex. Our data reveal that the pattern of mature miR166 accumulation results, in part, from intricate transcriptional regulation of its precursor loci and that only a subset of *mir166* family members contribute to the establishment of leaf polarity. We show that miR390, an upstream determinant in leaf polarity whose activity triggers *tas3* ta-siRNA biogenesis, accumulates adaxially in leaves. The polar expression of miR390 is established and maintained independent of the ta-siRNA pathway. The comparison of small RNA localization data with the expression profiles of precursor transcripts suggests that miR166 and miR390 accumulation is also regulated at the level of biogenesis and/or stability. Furthermore, *mir390* precursors accumulate exclusively within the epidermal layer of the incipient leaf, whereas mature miR390 accumulates in sub-epidermal layers as well. Regulation of miR390 biogenesis, stability, or even discrete trafficking of miR390 from the epidermis to underlying cell layers provide possible mechanisms that define the extent of miR390 accumulation within the incipient leaf, which patterns this small field of cells into adaxial and abaxial domains via the production of *tas3*-derived ta-siRNAs.

## Author Summary

Small RNAs regulate many key developmental processes. Consistent with a prominent role in development, miRNAs exhibit complex and distinctive expression patterns. In this study, we identify regulatory mechanisms that allow for the precise spatial accumulation of developmentally important small RNAs in plants. Plants generate new leaves throughout their lifetime. These arise on the flank of a specialized stem cell niche, termed *meristem*, at the plant's growing tip. Each newly formed leaf becomes polarized and develops distinct adaxial (top) and abaxial (bottom) sides. The establishment of adaxial–abaxial polarity requires a complex genetic network, including miRNAs and *trans*-acting siRNAs. We used a focused laser to microdissect regions of the shoot apical meristem and developing leaves of maize to analyze the expression profiles of the small RNA precursor molecules. By comparing these expression profiles to the accumulation patterns of the mature small RNAs, we show that precursor genes are subject to tissue-specific regulation and exhibit diverse expression patterns during leaf development. Our findings suggest that mechanisms exist to regulate the biogenesis, stability, and possibly even the intercellular movement of small RNAs. Such regulation should be considered when designing artificial miRNAs and has implications for the roles miRNAs play during plant and animal development.

## Introduction

Small regulatory RNAs play fundamental roles in diverse aspects of animal and plant development [1]–[2]. However, few examples exist in which small RNAs direct early patterning events. Leaves provide a unique developmental system in which multiple small RNAs pattern the adaxial-abaxial (dorsoventral) axis [3]. Leaf primordia arise as a group of determinate founder cells on the flank of the shoot apical meristem (SAM), a specialized stem cell niche at the growing tip of the shoot. The establishment of adaxial-abaxial polarity occurs early in leaf development and is concomitant with outgrowth of the primordium. Although polarity in leaves is ultimately specified through positional signals that convey to organ initials their proximity to the meristem tip [4], in maize the polarized expression of small RNAs, both microRNAs (miRNAs) and *trans*-acting siRNAs (ta-siRNAs), in the incipient leaf directs the patterning of the adaxial-abaxial axis [3].

MiRNA miR166 promotes abaxial/ventral fate in developing primordia by restricting the expression of class III homeodomain leucine zipper (HD-ZIPIII) transcription factors, which are necessary and sufficient to specify adaxial/dorsal fate [5]–[7]. Interestingly, the abaxial-restricted accumulation of miR166 is regulated by the *tas3* ta-siRNA pathway. Loss-of-function mutations in *leafbladeless1* (*lbl1*) that disrupt ta-siRNA biogenesis give rise to an abaxialized leaf phenotype and loss of *hd-zipIII* expression, demonstrating that the ta-siRNA pathway is necessary for specifying adaxial fate in maize [3],[8]. Accordingly, tasiR-ARFs, *tas3*-derived ta-siRNA species conserved between maize and *Arabidopsis*, accumulate on the adaxial side of incipient and developing leaf primordia, where they act to spatially restrict the expression domains of abaxial determinants. This includes, although indirectly, miR166, which is expressed ectopically in *lbl1* leaf primordia and accumulates in both the adaxial and abaxial domains [3]. Thus, organ polarity in leaves is ultimately achieved by regulating the spatial accumulation of both *tas3*-derived ta-siRNAs and miR166, which divide the small field of cells of the incipient primordium into adaxial and abaxial domains.

The functional importance of tasiR-ARF and miR166 activity in specifying the adaxial and abaxial domains of the leaf suggests mechanisms exist to maintain the accuracy of their spatiotemporal localization. However, even though our knowledge regarding small RNA biogenesis and function has increased significantly, and the detailed expression patterns of several mature miRNAs have been described [7], [9]–[11], little is known about such regulatory mechanisms. The biogenesis of these different small RNA classes in plants relies on specialized RNAi pathways. MiRNAs are ∼21 nucleotide small RNAs that arise from DICER-LIKE1 (DCL1)-dependent processing of precursor transcripts that contain a stem-loop structure. The mature miRNA forms a complex with ARGONAUTE1 (AGO1), creating an RNA-induced silencing complex (RISC) that silences transcripts with complementary target sites, predominately through cleavage [2]. In contrast, the biogenesis of ta-siRNAs is more complex, relying on both miRNA and siRNA pathway components. Following miRNA cleavage, ta-siRNA precursor (*tas*) transcripts enter into an RNA-DEPENDENT RNA POLYMERASE6 (RDR6) and LEAFBLADELESS1/SUPRESSOR OF GENE SILENCING3 (LBL1/SGS3)-dependent pathway and are processed by DICER-LIKE4 (DCL4) into phased, 21 nucleotide ta-siRNAs [12]–[15]. Although all ta-siRNA biogenesis follows this generic pathway, ta-siRNAs derived from the *TAS3* precursor family are processed using a subspecialized pathway dependent on miR390 activity. MiR390 forms a specialized RISC with AGO7/ZIP, the activity of which is required to recognize the 5′ and cleave the 3′ miR390 target sites of *TAS3A* precursor transcripts in *Arabidopsis* to produce *TAS3*-derived ta-siRNAs [16].

Post-transcriptional mechanisms that operate at the level of biogenesis could modulate the spatiotemporal localization of small RNAs, perhaps through the limited availability of necessary biogenesis factors, as has been proposed for animals [17]. In this respect, the diversity of specialized RNAi pathways in plants, as exemplified by the unique association of miR390 with AGO7/ZIP [16], would be one means to differentially regulate the accumulation of specific small RNA classes. Undoubtedly, transcriptional regulation of small RNA precursor transcripts also provides a mechanism by which the accumulation of mature small RNAs can be specified [18]–[19]. However, the analysis of the regulatory mechanisms that create precisely defined mature miRNA expression patterns in plants is hindered by the extensive redundancy present in miRNA precursor (*mir*) families, in which multiple *mir* genes produce identical mature miRNA species [2].

Here, we employ laser-microdissection (LM) to investigate the complex regulatory mechanisms that allow for the precise spatial accumulation of miR166 and small RNAs in the *tas3* ta-siRNA pathway in the maize SAM. The comparison of mature small RNA in situ localization data with the expression profiles of precursor transcripts, as determined using laser-microdissected domains, indicates that the accumulation pattern of mature miR166 results from the co-operative activities of multiple *mir* gene family members and is further regulated at the level of biogenesis and/or stability. Furthermore, we show that the polarized expression of miR390, an upstream component in leaf polarity, is established and maintained independently of the ta-siRNA pathway. The discrete adaxial accumulation of miR390 in the incipient leaf is regulated at the post-transcriptional level and might possibly result from limited mobility of mature miR390 over the span of a few cells.

## Results/Discussion

### Distinct *mir166* Expression Profiles Revealed by Laser-Microdissection

Mature miR166 exhibits a complex spatiotemporal pattern of expression. It is most abundant in a group of cells below the incipient leaf but a gradient of miR166 expression, which establishes organ polarity, extends into the abaxial side of the newly initiating primordium. During primordium development, miR166 expression persists on the abaxial side of the leaf. MiR166 also accumulates in the vascular bundles, specifically in the abaxial phloem [3],[7]. The maize genome contains at least nine *mir166* loci, *mir166a* through *mir166i*, which have the potential to produce identical mature miR166 species (miRbase release 10.1). To determine which family members contribute to the spatiotemporal expression pattern of mature miR166 in vegetative tissues, we investigated *mir166* gene expression in hand-dissected apices containing the SAM and four to five leaf primordia. Precursor transcripts of all nine *mir166* genes accumulate in vegetative apices (Figure S1). Similarly, all nine *mir166* genes are expressed in inflorescence tissues. This result could indicate substantial redundancy in the *mir166* gene family, or alternatively, *mir166* genes may have distinct, sublocalized expression patterns in these tissues.

To distinguish between these possibilities, we sought to localize individual *mir166* precursor transcripts in discrete domains of the maize apex. Plant miRNA precursors are typically low in abundance, presumably due to their rapid processing into mature miRNAs [2]. Consequently, expression analysis of *mir* genes using in situ hybridization is often not successful [3],[7]. We therefore employed laser-microdissection (LM) [20]–[21] coupled to reverse-transcriptase PCR (LM-RT-PCR) as a technique to detect *mir166* transcripts at cellular resolution [3]. The comparative analysis of *mir* precursor localization as determined by LM-RT-PCR in combination with localization of mature miRNAs is a potentially powerful technique to reveal novel mechanisms regulating the accumulation of miRNAs.

**Figure 1:**
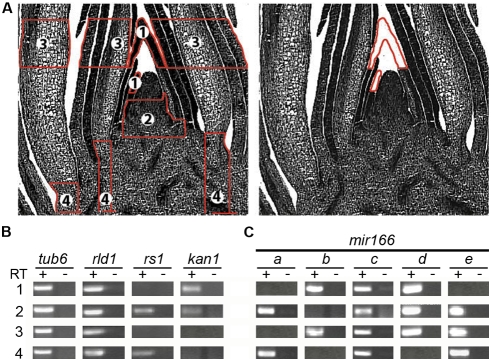
*mir166* family members exhibit distinct overlapping expression profiles in the maize shoot apex. (A) Longitudinal sections through a maize apex diagramming regions captured by laser-microdissection (left panel): 1) P2–P3 leaf primordia; 2) the incipient (P0) and P1 leaf primordia plus the base of the SAM; 3) P4–P6 leaf primordia; 4) stem tissue. The right panel shows a longitudinal section of an apex after capturing cells from the P2 and P3 leaf primordia. Note the precision with which cells are captured. (B) Transcript accumulation of *tub6*, *rld1*, *rs1* and *kan1* in these regions is as previously reported [7], [22]–[23], illustrating the accuracy of laser-microdissection. (C) RT-PCR analysis of *mir166a* to *mir166e* shows that these *mir166* family members exhibit distinct but overlapping expression profiles within the vegetative apex. -RT controls are also shown.

As an initial analysis, we investigated the expression of a subset of *mir166* genes in relatively broad domains of the maize apex (Figure 1). Cells were captured from regions in which miR166 accumulates [7], including: 1) the P2 and P3 developing leaf primordia; 2) the incipient leaf (P0) where polarity is established, the P1, and the region of tissue just below the SAM; 3) more developed P4-P6 primordia; and 4) stem tissue, which contains extensive vasculature (Figure 1A). The expression profile of selected control genes in the microdissected domains was tested to determine the accuracy of LM. Consistent with their reported in situ hybridization expression patterns, the miR166 target *rolled leaf1* (*rld1*) is expressed in all tissue samples tested [7], *rough sheath1* (*rs1*) transcripts are limited to those domains that include the subtending regions of leaves [22], and similar to *kanadi2* (*kan2*), the expression of *kan1* demarks cells in the developing young leaf primordia [23] (Figure 1B).

Although multiple *mir166* family members are expressed in every tissue sample analyzed, each *mir166* gene tested exhibits a unique expression profile (Figure 1C). For instance, *mir166a* precursor transcripts are detected in the SAM region and stem, whereas transcripts from *mir166b* accumulate in P2 and older leaf primordia (Figure 1C). *mir166c*, -*d*, and -*e* are expressed more broadly throughout the apex, but each still displays a distinct expression profile. The LM-RT-PCR approach thus allows analysis of maize *mir* gene expression with both high sensitivity and spatial resolution, without relying on transgenic approaches. The data also indicates that the complex pattern of miR166 accumulation results in part from the distinct transcriptional regulation of individual *mir166* precursors. The partially overlapping expression patterns of *mir166* precursors suggest some degree of redundancy. However, the expression profiles of individual *mir166* genes are unique, indicating a process of functional diversification perhaps not unlike their *hd-zipIII* targets, which have distinct but overlapping roles in organ polarity, vascular development and meristem activity [24]–[25]. For example, their expression in stem tissues suggests a possible function for *mir166a*, -*c*, and -*e* in vascular differentiation (Figure 1C). Similarly, the accumulation of *mir166b* transcripts in P2 and older leaf primordia may indicate a role in maintaining, rather than establishing, leaf polarity.

### Specific *mir166* Family Members Establish Leaf Polarity

**Figure 2:**
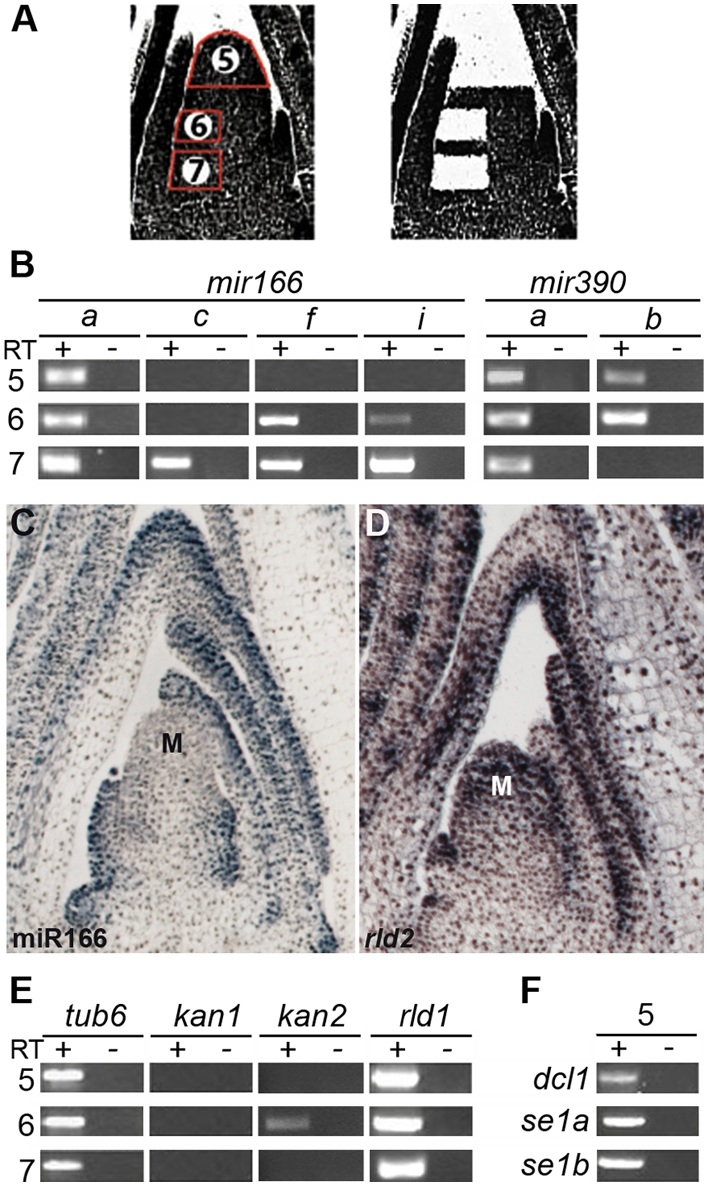
Accumulation of miR166 and miR390 within the SAM is regulated at the level of biogenesis and/or stability. (A) Laser-microdissection of domains within the SAM: 5) the stem cell containing SAM tip; 6) the incipient primordium; 7) the region below the incipient leaf. (B) Expression analysis of *mir166* family members by RT-PCR shows that only *mir166a*, -*c*, -*f* and –*i* precursor transcripts accumulate in or below the incipient leaf and likely contribute to the establishment of organ polarity. In addition, *mir166a* transcripts are expressed in the SAM tip. Whereas both *mir390* precursors are expressed in the tip of the SAM and in the incipient leaf, only *mir390a* accumulates in the region below the incipient leaf. (C) In situ hybridization of a longitudinal section through a wild-type apex with a miR166 complementary LNA probe shows miR166 accumulates below the incipient leaf and on the abaxial side of the incipient and developing leaf primordia. Note the lack of detectable signal in the tip of the SAM (marked “M”). (D) Longitudinal section through a wild-type apex shows *rld2* expression in the tip of the SAM, vasculature, and the adaxial side of leaf primordia. (E) Expression of *tub6*, *kan1*, *kan2*, and *rld1* in the captured SAM regions is as previously reported [7],[23], illustrating the accuracy of laser-microdissection. (F) RT-PCR analysis for the core miRNA processing genes *dcl1*, *se1a* and *se1b* in the SAM tip.

To determine which *mir166* genes act in the SAM to establish the abaxial-graded pattern of miR166 in the incipient leaf [3],[7] and therefore contribute to the specification of leaf polarity, we next microdissected precisely defined domains within the SAM. Expression of *mir166a* through *mir166i* was evaluated in cells captured from below the incipient leaf, the incipient primordium, as well as the tip of the SAM in which mature miR166 does not accumulate (Figure 2A). The expression profiles of the control genes *kan1*, *kan2* and *rld1* in these domains recapitulate their described expression patterns and thus verify the purity of the LM samples (Figure 2E). Transcripts of only a subset of *mir166* family members are detected in the SAM. *mir166a*, -*f* and -*i* are expressed both in and below the incipient primordium, whereas *mir166c* is expressed at detectable levels exclusively below the initiating leaf (Figure 2B). This data further supports the existence of functional diversification within the *mir166* gene family and shows that a subset of *mir166* precursor genes contributes to the abaxial accumulation of mature miR166 that is required for establishing abaxial fate.

**Figure 3:**
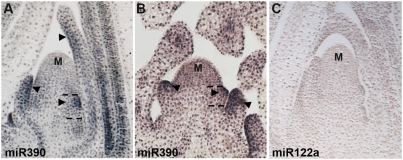
MiR390 is an upstream component in the maize leaf polarity pathway. (A, B) In situ hybridization analyses with a miR390 complementary LNA probe shows this small RNA accumulates on the adaxial side (arrowheads) of incipient and developing leaf primorida of both wild-type (A) and *lbl1-rgd1* (B) seedlings. Dashed lines mark the position of the incipient leaf. No hybridization signal is detected in the tip of the wild-type SAM. (C) In situ hybridization with an LNA-probe complementary to murine miR122a serves as a negative control.

Interestingly, *mir166a* precursor transcripts also accumulate in the tip of the SAM (Figure 2A and 2B). The detection of *mir166a* transcripts in this domain is surprising, considering that mature miR166 is not detectable in this region by in situ hybridization using either a riboprobe [3],[7] or a highly-sensitive LNA-based probe (Figure 2C). The abundance of *rld1* and *rld2* target transcripts in the meristem tip (Figure 2D and 2E; [3],[7]) further suggests a lack of miR166 activity in this region. The tip of the SAM contains a population of indeterminate stem cells. The lack of observable miR166 accumulation in this region raises the possibility that either the maturation and/or stability of this miRNA is compromised in these cells. Plant stem cells may lack essential components of the miRNA processing machinery. Although we can detect transcripts of *dcl1* and *se* homologs, core components of the miRNA precursor-processing pathway, in the tip of the SAM (Figure 2F), the possibility that other miRNA processing components are lacking cannot be excluded. Alternatively, selected miRNA precursors may fail to engage the processing apparatus in stem cells through a hitherto unknown inhibitory process. A similar accumulation of unprocessed precursor transcripts occurs in mammalian embryonic stem cells in which Lin28 inhibits the Drosha-mediated processing of differentiation-promoting miRNAs [17], [26]–[27]. Whether plant stem cells utilize an analogous mechanism to block the biogenesis of miRNAs associated with differentiation remains to be seen.

### MiR390 Accumulation is Adaxially Restricted in Incipient and Developing Leaves

The finding that *mir166c* and *mir166i* are expressed within and below the incipient leaf (Figure 2B) is consistent with previous observations showing that the *tas3* ta-siRNA pathway generates the abaxial gradient of miR166 and specifies adaxial fate by restricting the expression domains of these two specific *mir166* family members [3]. Maize tasiR-ARFs, which accumulate most pronounced on the adaxial side of developing leaf primordia, are produced from at least four *tas3* loci (*tas3a*-*tas3d*). To identify which *tas3* family members contribute to the localized accumulation of tasiR-ARFs in the incipient leaf, we analyzed the expression of *tas3a-d* precursors in the SAM. Unlike the *mir166* genes, all four *tas3* family members are expressed within and below the incipient leaf and thus likely contribute redundantly to the establishment of leaf polarity (Figure S2). Moreover, *tas3b-d* transcripts are expressed in the tip of the SAM where tasiR-ARFs do not accumulate. A key component of tasiR-ARF biogenesis, *lbl1/SGS3*, is expressed in the meristem tip as well as on the adaxial side of the incipient and developing leaves [3]. The restricted activity of additional *tas3* ta-siRNA biogenesis components therefore likely limits the accumulation of tasiR-ARFs to only the adaxial side of developing primordia and prevents their accumulation in the SAM tip.

As the biogenesis of *tas3* ta-siRNAs is uniquely triggered by miR390 activity [12], we determined the localization pattern of mature miR390 by in situ hybridization to ascertain whether it might be a restrictive component limiting tasiR-ARF biogenesis. Whereas an LNA probe against a murine miRNA yields no detectable hybridization signal, an LNA probe complementary to miR390 shows it accumulates adaxially within the incipient and developing leaves (Figure 3A and 3C). Additionally, miR390 is expressed in vascular bundles (Figure 3A). The adaxial accumulation of miR390 overlaps with that of tasiR-ARFs and the expression pattern of *lbl1/SGS3* in these regions [3], and is opposite to the abaxial accumulation of miR166 in the incipient leaf (Figures 2C and 3A). Thus, miR390 is a restricting factor in the *tas3* ta-siRNA pathway that limits the biogenesis of tasiR-ARFs to within developing leaves. That miR390 restricts the accumulation of tasiR-ARFs places it as one of the upstream factors in the maize leaf polarity network, as genetic and expression data position the *tas3* ta-siRNA pathway upstream of miR166 as well as the *hd-zipIII* and *yabby* genes [3],[8].

As a result of the mutually antagonistic relationship between the adaxial and abaxial sides of the leaf, the expression of adaxial and abaxial determinants, such as the *HD-ZIPIII*, *YABBY* and *KANADI* genes, changes in mutants with perturbed leaf polarity [4]. We therefore determined the pattern of miR390 expression in the apex of *lbl1-rgd1* mutants, which develop fully abaxialized, radially symmetric leaves [28]. Unexpectedly, miR390 remains polarized within the initiating and developing leaf primordia of *lbl1-rgd1* (Figure 3B), even though these leaves are molecularly abaxialized with respect to expression of miR166 and members of the *hd-zipIII* and *yabby* families [3],[8]. This persistent adaxial expression of miR390 in *lbl1* mutants further highlights the importance of this small RNA as an upstream component in the maize leaf polarity pathway, whose polarized expression is established independent of the ta-siRNA pathway.

To gain insight into the regulatory mechanisms that direct the accurate spatiotemporal localization of mature miR390 in the incipient leaf, we analyzed expression of its precursors using LM-RT-PCR. Previously, we had cloned a single *mir390* precursor [3]; however, analysis of the completed draft sequence of the maize genome revealed one additional *mir390* locus (Maize Genome Sequencing Consortium; http://www.maizesequence.org). The two *mir390* precursors, referred to hereafter as *mir390a* and *mir390b* (Figure S3), map to maize chromosomes one and five to regions of synteny with the rice and sorghum genomes containing a single *mir390* locus. Only *mir390a* is expressed below the incipient primordium, but both *mir390a* and *mir390b* are expressed in the initiating leaf and may act redundantly in the biogenesis of miR390 and specification of leaf polarity (Figure 2B). Similar to our observations for *mir166a*, both *mir390* precursor transcripts are detectable in the tip of the SAM, although mature miR390 does not accumulate in this domain (Figures 3A and 2B). This suggests that the post-transcriptional regulatory mechanism that limits the accumulation of miR166 in the stem cell-containing meristem tip also limits the accumulation of other miRNAs, perhaps acting as a general mechanism to limit miRNA-induced differentiation [17].

### Restriction of *mir390* Precursors to the L1 Layer of the SAM

**Figure 4:**
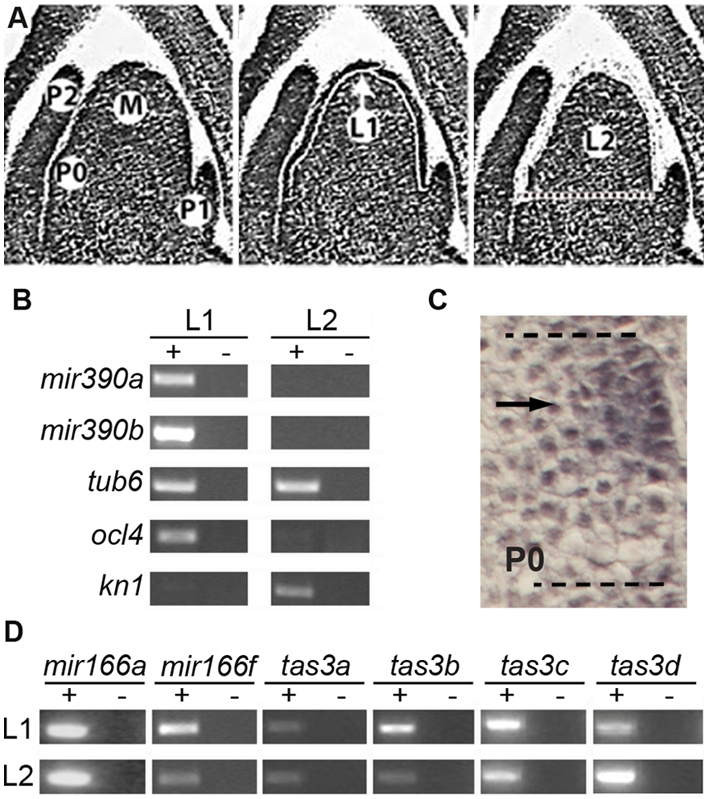
*mir390* precursors, unlike mature miR390, accumulate in just the epidermal (L1) layer of the maize SAM. (A) Representative sections showing the laser-microdissection of the L1 epidermal layer of the SAM (M) including the incipient primordium (P0). The L1 layer is precisely cut (middle panel) and subsequently captured (right panel). Afterwards, the L2 layer is microdissected similarly. (B) RT-PCR analysis of the miR390 precursors shows that both *mir390a* and *mir390b* transcripts accumulate specifically in the L1 layer. Mutually exclusive expression of *ocl4* and *kn1* in the L1 and L2 layers, respectively, demonstrates the precision of LM [22],[31]. (C) Close-up view of the accumulation pattern of mature miR390 in the incipient leaf (dashed lines) taken from Figure 3A. Note the presence of signal in the underlying L2 layer (arrow). (*D*) RT-PCR analysis of *mir166* and *tas3* family members shows these precursor transcripts accumulate in both the L1 and L2 layers of the SAM.

Surgical experiments separating the initiating leaf from the meristem produce radially symmetric, abaxialized leaves, suggesting the existence of a meristem-borne signal that establishes adaxial fate [29]. More recently, precise microsurgery experiments in tomato have shown that incisions in just the epidermal cell layer (L1) similarly disrupt adaxial-abaxial patterning of leaf primordia [30], suggesting that the L1 layer is necessary for the adaxial-promoting signal to function. As miR390 constitutes an upstream component in the maize leaf polarity network, we compared the accumulation of *mir390* precursor transcripts between the L1 and sub-epidermal L2 layers of the SAM (Figure 4A). Both *mir390* primary transcripts accumulate exclusively within the L1 layer (Figure 4B). This is contrary to the accumulation of mature miR390, which despite the decreased sensitivity of in situ hybridization as compared to LM-RT-PCR, is detectable at least three cell layers internal to the incipient leaf's surface (Figure 4C). The accuracy of LM is demonstrated through the mutually exclusive expression of the tissue specific markers *outer cell layer4* (*ocl4*) in the L1 layer and *knotted1* (*kn1*) in the L2 layer (Figure 4B; [22],[31]). Furthermore, the inability to detect *mir390* precursor transcripts in the L2 samples is unlikely an artifact of sample dilution effects resulting from the fact that the L2 includes more cells than the L1 layer. Microarray analysis of the exact samples used in this study shows comparable expression levels in the L1 and L2 samples for the leaf polarity determinants *rld1*, *rld2*, *Zmyabby9*, and *Zmyabby14*, all of which are known to be expressed in both the L1 and L2 layers of the incipient leaf ([8] (K. Ohtsu and P. Schnable, unpublished data). Likewise, *tubulin6*, *mir166a*, *-f*, and all *tas3* transcripts were detectable in both the L1 and L2 layers (Figure 4B and 4D).

As cells in the L1 layer divide almost exclusively anticlinally, accumulation of miR390 in the sub-epidermal layers does not simply reflect inheritance from dividing epidermal cells in the incipient primordium. The non-overlapping localization patterns of mature miR390 and its precursor transcripts could reflect increased processing of miR390 in the L2 layer compared to the L1 layer. However, assuming that the expression of *mir390* transcripts before their processing is approximately equal in the L1 and L2 layers, miR390 would be expected to accumulate most abundantly in the L2, as no precursor transcripts accumulate there. However, in situ hybridization shows mature miR390 is equally or less abundant in the underlying L2 as compared to the L1 (Figure 4C). The observed incongruence between the expression patterns of mature miR390 and the *mir390* precursors could conceivably also reflect mobility of the miR390 small RNA from the epidermis into the underlying cells. Although miRNAs are thought to act largely cell autonomously [32]–[34], mobility over just a few cells or in specific developmental contexts remains a possibility. Given that no maize mutants impairing miRNA biogenesis have been described, we cannot currently distinguish between these possibilities.

### Establishment of Maize Leaf Polarity via Small Regulatory RNAs

That miR390 remains polarized in the abaxialized leaves of *lbl1-rgd1* mutants (Figure 3) and is responsible for restricting tasiR-ARF accumulation to the adaxial side of leaves places this small RNA upstream in the maize leaf polarity pathway [3],[8]. The restricted accumulation of miR390 demarcates the adaxial most cells within developing leaves that produce *tas3*-derived ta-siRNAs, which in turn restrict the accumulation of miR166 to the abaxial side of leaf primordia [3]. As an upstream initiator of a small RNA cascade that ultimately patterns the leaf into adaxial and abaxial domains, the extent of miR390 activity must be precisely defined. Such precision is likely achieved through a variety of mechanisms regulating miRNA biogenesis, stability, and possibly movement. Both *mir390* genes are expressed outside the incipient leaf, in the L1 layer of the entire SAM, as their precursors are detected in microdissected samples that comprise just the tip of the shoot apex or the L1 layer, but not in samples comprising the L2 layers (Figure 4A and 4B). *mir390a* is also expressed below the incipient leaf. Nevertheless, regulation of miR390 biogenesis and/or stability allows this small RNA to accumulate only in the few adaxial cells of the incipient primordium and not elsewhere in the SAM, such as in the tip (Figure 3A). Although the mechanisms for such regulation are not currently understood, these should function independent of the ta-siRNA pathway, as miR390 remains localized to the incipient primordium in *lbl1-rgd1* (Figure 3B). The post-transcriptional regulatory mechanisms that limit miR390 accumulation in the SAM tip may similarly regulate other miRNAs such as miR166, which is not detected in the meristem despite the presence of precursor transcripts (Figure 2). The restricted adaxial accumulation of miR390 in the incipient leaf might also be achieved through channeling into a subspecialized RNAi pathway. MiR390 is selectively incorporated into an AGO7/ZIP complex to execute the processing of *tas3* precursor transcripts [16]. Such an association with AGO7/ZIP, or other components of this unique RNAi branch, might selectively stabilize miR390 on the adaxial side of the leaf.

**Figure 5:**
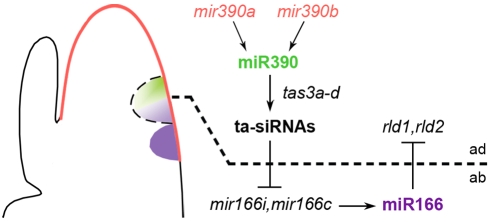
A model for the establishment of maize leaf polarity. The diagram on the left illustrates the spatial pattern of expression for both *mir390* precursors as well as mature miR390 and miR166 in the SAM. The *mir390* precursors accumulate in the L1 layer of the whole SAM (red). In contrast, mature miR390 (green) is present in both the L1 and underlying L2 layers, but just on the adaxial side of the incipient leaf. The adaxial expression of miR390 persists even in the abaxialized leaves of *lbl1* mutants, demonstrating that this small RNA, which constitutes an upstream component in the maize adaxial-abaxial patterning network, maintains polar expression independent of the *tas3* ta-siRNA pathway. MiR390 activity triggers ta-siRNA biogenesis on the adaxial side of the incipient primordium that indirectly restrict the expression of specific *mir166* family members. Regulation of small RNAs in the ta-siRNA pathway at the level of biogenesis, stability, or possibly movement generates an inverse abaxial gradient of mature miR166 (purple) that delineates the expression of *hd-zipIII* genes, like *rld1* and *rld2*, to the adaxial side of the incipient leaf establishing organ polarity.

Besides such regulatory mechanisms that limit the biogenesis and/or stability of miR390, discrete trafficking from the epidermis into underlying cell layers could potentially provide an additional mechanism that defines the extent of miR390 activity within the incipient leaf. The hypothesis of miRNA movement from the L1 epidermal layer is intriguing, as it provides an appealing explanation for miR390's upstream role in defining the boundary between the adaxial and abaxial domains of leaves, and is consistent with an earlier hypothesis suggesting that mobility of ta-siRNA pathway components may generate the abaxial graded pattern of miR166 accumulation [3]. The movement of miR390 could feasibly result in a pattern of graded activity that would translate into a gradient of *tas3* ta-siRNA accumulation from the adaxial side of the leaf. As *tas3* ta-siRNAs indirectly restrict the expression of *mir166c* and *mir166i*, such a ta-siRNA gradient could specify the inverse graded accumulation of miR166 on the abaxial side of the leaf that adaxially restricts *hd-zipIII* transcripts (Figure 5). In addition to miR390, non-cell autonomy of ta-siRNAs remains a possibility, as ta-siRNAs are processed by DCL4 and RDR6, factors required for the biogenesis of mobile siRNAs during systemic silencing in *Arabidopsis* [35]–[36].

### Conclusion

Our findings indicate that small amounts of RNA from laser-microdissected tissue samples are sufficient to detect *mir166* and *mir390* precursors, providing a novel approach to study miRNA regulation in specific cell-types and stages of development in plants and, perhaps, animals. Along with in situ hybridization analyses, our results suggest that miR166 and miR390 accumulation is subject to complex tissue and cell-type specific transcriptional and post-transcriptional controls, allowing for functional diversification of miRNA gene family members in development. The potential limited movement of miR390 has significant implications for the possible roles of small RNAs during development in establishing patterning events. Finally, the complex mechanisms regulating the biogenesis, stability, and possible movement of miRNAs should be taken into consideration when designing artificial miRNA studies and interpreting the intricate miRNA accumulation patterns in plants and animals.

## Materials and Methods

### Sequence Analysis

Sequences corresponding to the *mir166a* to *mir166i* precursors were retrieved from miRbase (http://microrna.sanger.ac.uk/sequences) and extended using maize EST and genome sequence databases (http://magi.plantgenomics.iastate.edu and http://www.maizesequence.org). The *mir390a* precursor has been described previously [3]. Sequences for *mir390b* were assembled using the above-mentioned databases, and the BAC accession number for *mir390b* is AC197125. The maize genome contains at least five *dcl* genes; two most similar to *Arabidopsis* DCL3 and one each most similar to DCL1, DCL2, and DCL4 (Figure S4; http://www.chromdb.org). The BAC accession number for *dcl1* is AC191256. The maize *SERRATE* homologs have been described previously [37]. Secondary structures for the *mir390a* and *mir390b* precursors were predicted using RNAfold.

### RNA Isolation and Analysis

Total RNA was extracted from vegetative apices including the SAM and four to five leaf primordia and from immature ears (0.5–1 cm) using TRIzol reagent (Invitrogen) according to the manufacturer's instructions. DNaseI-treated RNA was converted into first-strand cDNA and amplified by PCR according to standard protocols. Primer sequences used in this study are listed in Table S1.

### In Situ Hybridization

Tissue sections prepared from the shoot apices of two-week-old wild-type or *lbl1-rgd1* seedlings were pretreated and hybridized as previously described [38]. LNA probes with sequences complementary to miR390, miR166, and murine miR122a were synthesized by Exiqon (Vedbaek, Denmark) and digoxigenin-labeled (Roche) according to the manufacturer's protocol. Ten picomoles of each probe were used per slide pair and hybridization and washing steps were performed at 50°C. The *rld2* probe, comprising nucleotides 625–1677 of the coding sequence, was used at a concentration of 0.5 ng/µL/kb as described [3].

### Laser-Microdissection and Reverse Transcriptase (RT)-PCR

Shoot apices from two-week old wild-type seedlings were dissected and immediately fixed in pure, ice-cold acetone. Acetone was gradually replaced with xylene and subsequently paraplast. Cells of interest were captured from 10 µm tissue sections using a PALM MicroBeam system. Detailed embedding and LM protocols have been described previously [20],[21]. Total RNA was extracted and linearly amplified using PicoPure RNA Isolation and RiboAmp HS RNA Amplification Kits (Arcturus Bioscience, Inc.) according to the manufacturer's protocols. RNA was treated with DNaseI and subsequently analyzed by RT-PCR using a one-step method (Qiagen) and the primers listed in Table S1. Three biological replicates with at least two technical replicates were performed.

## Supporting Information

Figure S1 Expression analyses of *mir166* family members in maize vegetative apices and female inflorescence tissues. (A) RT-PCR amplification of *mir166a - i* precursor transcripts on total RNA isolated from hand-dissected vegetative apices and female inflorescences (∼0.5–1 cm) shows that all nine *mir166* genes are expressed in both tissues. The loading control *tubulin6 (tub6)* and -RT controls are shown. (B) Cartoon representing a *mir166* precursor transcript with the miRNA* (blue) and mature miRNA (red) shown. Gene specific primers (arrows) were designed downstream of the stem-loop as indicated.
(0.15 MB JPG)

Figure S2 *tas3* genes are expressed broadly throughout the maize shoot apical meristem. Cells were captured by laser-microdissection from the tip of the SAM (5), the incipient leaf (6) and below the incipient leaf (7) as depicted in Figure 2 of the manuscript. mRNA isolated from these microdissected domains was linearly amplified and used in 1-step RT-PCR to monitor expression of tas3 family members. The *tas3a - tas3d* precursors are expressed broadly throughout the SAM, and only *tas3a* does not appear to be expressed in the tip of the SAM.
(0.07 MB JPG)

Figure S3 Diagrams showing the sequences and secondary structure of the *mir390a* (A) and *mir390b* (B) precursor stem-loops. Blue lines mark the mature miR390 sequence.
(0.17 MB JPG)

Figure S4 Phylogenetic analysis of DICER-LIKE (DCL) proteins from maize, rice and Arabidopsis identifies a single conserved maize DCL1 protein. (A) Un-rooted phylogram of *Arabidopsis* (At), rice (Os) and maize (Zm) DICER-LIKE proteins. Four sub-groups comprising DCL1, DCL2, DCL3 and DCL4 homologs are distinguished within the phylogenetic tree. The phylogram was generated as a consensus of 1000 bootstrap replicates by the neighbor joining method using the MEGA2 software. The scale bar indicates the relative frequency of changes along the branches. At least five dcl genes have been identified in maize; two most similar to *Arabidopsis DCL3* and one each most similar to *DCL1*, *DCL2*, and *DCL4*. Protein sequences were retrieved from the chromatin database (http://www.chromdb.org). (B) ZmDCL1 (bold in A) contains conserved domains characteristic of the DCL proteins belonging to the *Arabidopsis* DCL1 sub-group. Protein domains were predicted using Pfam (http://pfam.wustl.edu/).
(0.28 MB JPG)

Table S1 Sequences of primers used in this study.
(0.05 MB DOC)
