# Supplementary figures and images for: Regulation of Small RNA Accumulation in the Maize Shoot Apex
Source: PLoS Genet. 2009 Jan 2;5(1):e1000320. doi: 10.1371/journal.pgen.1000320 (PMC2602737; doi:10.1371/journal.pgen.1000320)

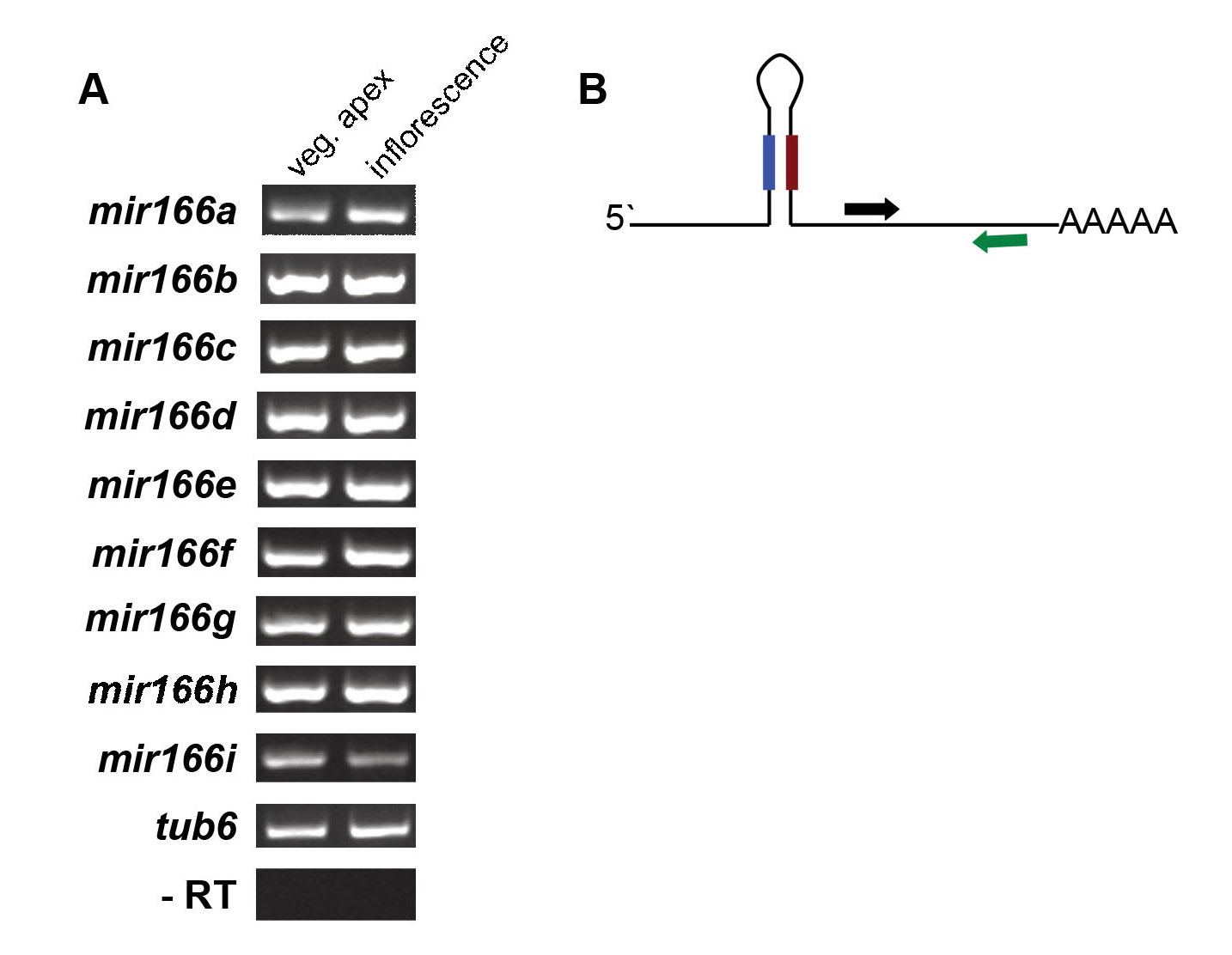

Supplement: Figure S1 — Expression analyses of mir166 family members in maize vegetative apices and female inflorescence tissues. (A) RT-PCR amplification of mir166a - i precursor transcripts on total RNA isolated from hand-dissected vegetative apices and female inflorescences (∼0.5–1 cm) shows that all nine mir166 genes are expressed in both tissues. The loading control tubulin6 (tub6) and -RT controls are shown. (B) Cartoon representing a mir166 precursor transcript with the miRNA* (blue) and mature miRNA (red) shown. Gene specific primers (arrows) were designed downstream of the stem-loop as indicated. (0.15 MB JPG) [file pgen.1000320.s001.jpg]

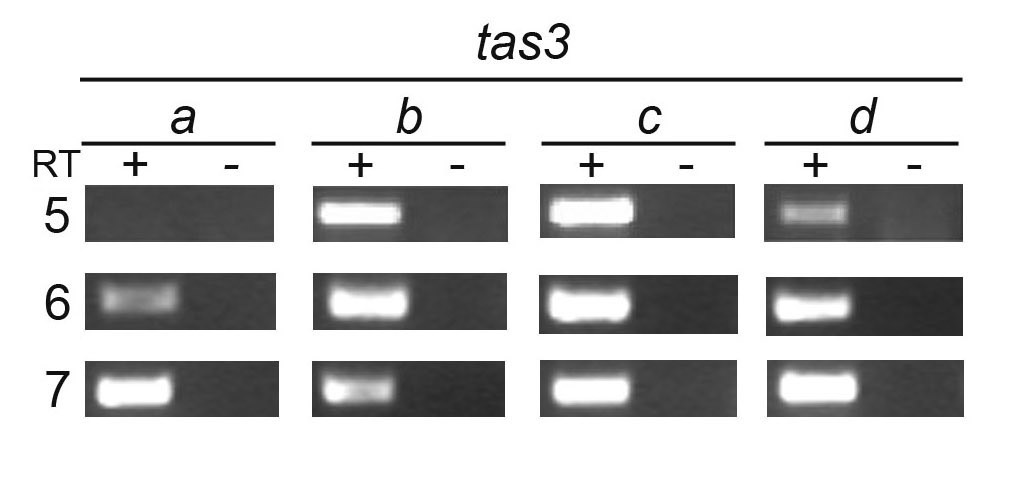

Supplement: Figure S2 — tas3 genes are expressed broadly throughout the maize shoot apical meristem. Cells were captured by laser-microdissection from the tip of the SAM (5), the incipient leaf (6) and below the incipient leaf (7) as depicted in Figure 2 of the manuscript. mRNA isolated from these microdissected domains was linearly amplified and used in 1-step RT-PCR to monitor expression of tas3 family members. The tas3a - tas3d precursors are expressed broadly throughout the SAM, and only tas3a does not appear to be expressed in the tip of the SAM. (0.07 MB JPG) [file pgen.1000320.s002.jpg]

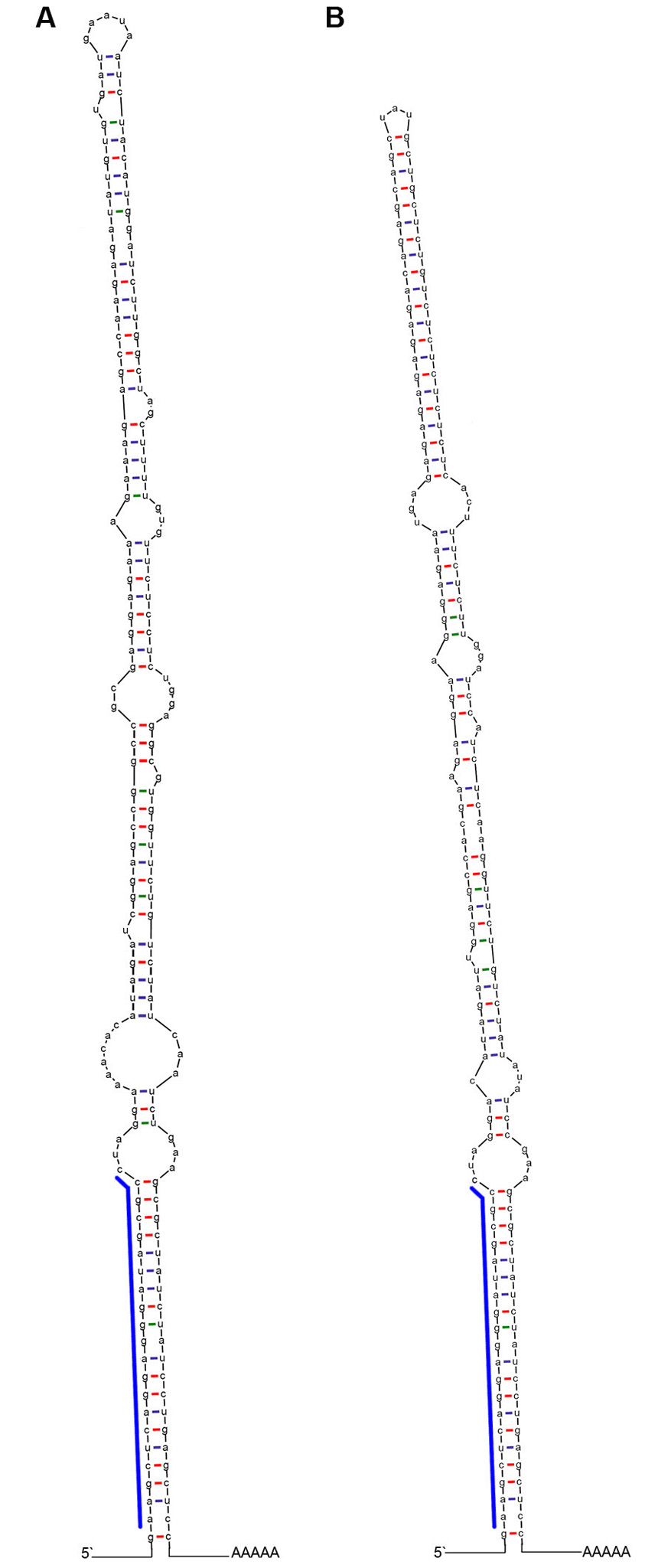

Supplement: Figure S3 — Diagrams showing the sequences and secondary structure of the mir390a (A) and mir390b (B) precursor stem-loops. Blue lines mark the mature miR390 sequence. (0.17 MB JPG) [file pgen.1000320.s003.jpg]

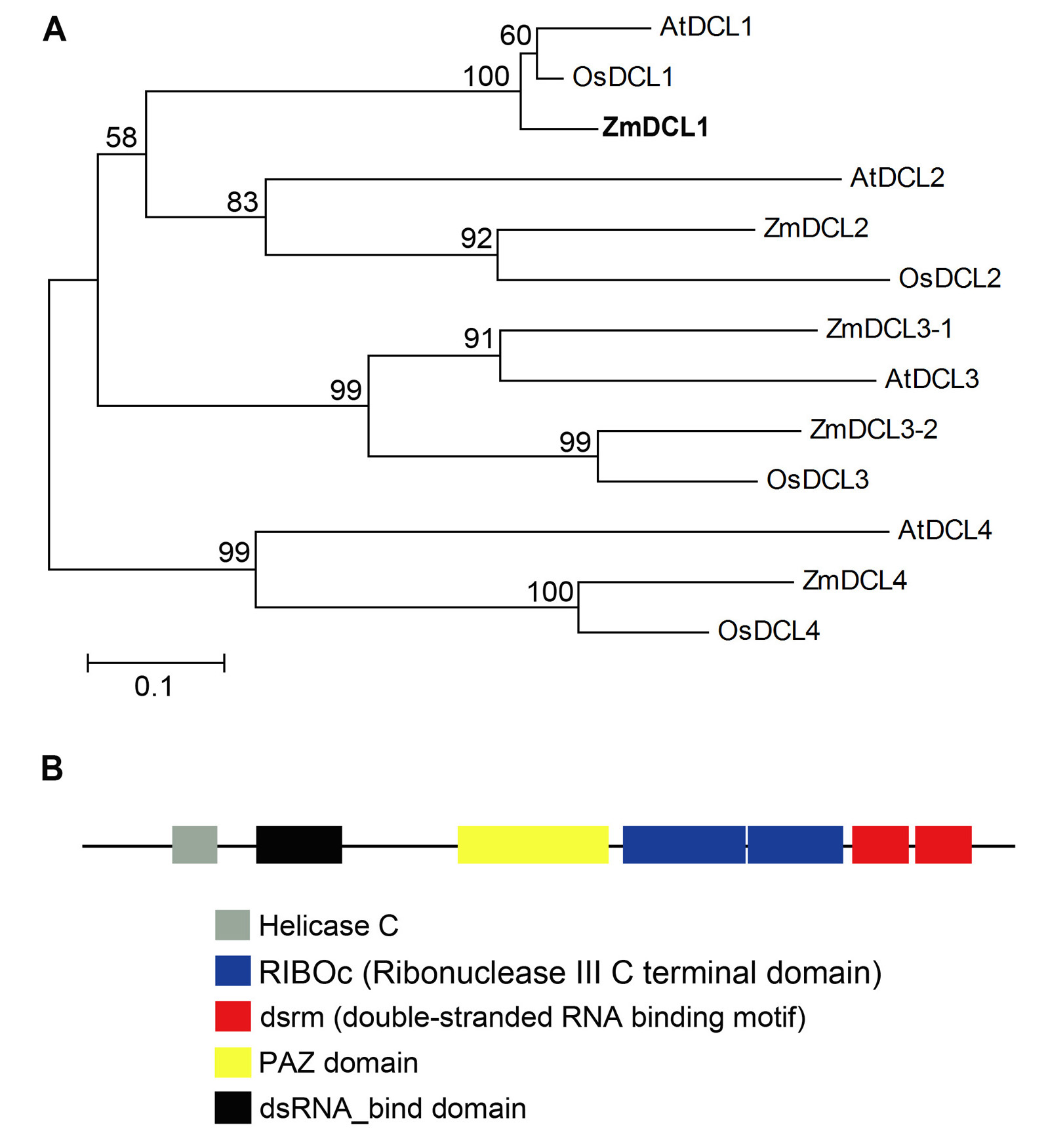

Supplement: Figure S4 — Phylogenetic analysis of DICER-LIKE (DCL) proteins from maize, rice and Arabidopsis identifies a single conserved maize DCL1 protein. (A) Un-rooted phylogram of Arabidopsis (At), rice (Os) and maize (Zm) DICER-LIKE proteins. Four sub-groups comprising DCL1, DCL2, DCL3 and DCL4 homologs are distinguished within the phylogenetic tree. The phylogram was generated as a consensus of 1000 bootstrap replicates by the neighbor joining method using the MEGA2 software. The scale bar indicates the relative frequency of changes along the branches. At least five dcl genes have been identified in maize; two most similar to Arabidopsis DCL3 and one each most similar to DCL1, DCL2, and DCL4. Protein sequences were retrieved from the chromatin database (http://www.chromdb.org). (B) ZmDCL1 (bold in A) contains conserved domains characteristic of the DCL proteins belonging to the Arabidopsis DCL1 sub-group. Protein domains were predicted using Pfam (http://pfam.wustl.edu/). (0.28 MB JPG) [file pgen.1000320.s004.jpg]
