# Supplementary material for: Regulation of Small RNA Accumulation in the Maize Shoot Apex
Source: PLoS Genet. 2009 Jan 2;5(1):e1000320. doi: 10.1371/journal.pgen.1000320 (PMC2602737; doi:10.1371/journal.pgen.1000320)
Supplement: Table S1 — Sequences of primers used in this study. (0.05 MB DOC) [file pgen.1000320.s005.doc]

| **Gene** | **Forward (5`->3`)** | **Reverse (5`->3`)** | **Nested (5`->3`)** |
| --- | --- | --- | --- |
| *mir166a* | GGGTACGTACATGCAAATCTGA | TCCTCCTCTCTTCAGTCGTCTT | TTCCTACTCCTATCTCGATCCC |
| *mir166b* | GTTGTCTGGTTCAAGGTCTTGCT | TACACCAATCTTGCTGCTCCT | TCCGATTTGAGGATGATCCATGC |
| *mir166c* | GTGCAATCATAAGGATTCTTGACC | GATAGCGATGATACACAACCTTTTC | GGTCGTCTTGGTTGTTGTTGATC |
| *mir166d* | TGCTTTGTGATCCATCTGTGTAG | ATTTCGCAGCAAGCAAGCAAG | GTGTAGAGCTTCTAAGTATTCCTTGGATCT |
| *mir166e* | TCTCATCGGTCTAGGTTCTTGTC | CAAGCATGCCTGTGTGTTCTT | CTGTTCTTGCCGTGAGTTTCC |
| *mir166f* | CCAGAGAGGACTCAGAAAGAGATG | AGCGCCACACGTAATAAGAAAG | ACGATTCACCATGGAAACGG |
| *mir166g* | GCATGATGGTGGAAGGTTTC | CAGTATCAAGGAGCGAGTGC | TGCATCTATCCATGCATGATGGT |
| *mir166h* | TGAAGCGCTACTCTGCTACG | CACACACATTTCAGGGTCCA | ACTCATGGAGACGGCAGTGATCC |
| *mir166i* | CTTCCAAGATTGCTTTGTCAGTCGT | TGCTATATGAAACGTACAAACTATTCG | ACGTACAAACTATTCGCTTGCGTTC |
| *mir390a* | CAAGCGATGAGGTGAGGTCTG | GGTGGTTTAGTCAGTCAGTTACATTC | - |
| *mir390b* | TGTGTGCGTCGTACTCCTTC | GATAACACGTGCGACTGGTG | - |
| *tas3a* | GTAAGGCCTCTTCTTGACCTTGTA | CACAGGGTGAAAACATTAACTGAAC | - |
| *tas3b* | CTTGACCTTGTAAGACCCAACTCTA | TGTTTGTCTCATGCCTCACTCTAT | - |
| *tas3c* | GGTTCACGTGGTTCATGTCTAGTAT | AAACATATAGTTGAACCCACAGCTC | - |
| *tas3d* | GGTTTCTCGTGCCAGAATTAAC | AATAATTTCAACGCCACCAAAC | - |
| *dcl1* | TCTGCTTGAGAAGGCAATGG | ACCACCACAGAACATCACCA | - |
| *se1a* | TGCATGATCCTCGCAGAATA | AGTGGTCGCGACACAAAAAG | - |
| *se1b* | TTGTAGGCTTGCCTCCAGAT | TGCTCAAACATGGACGAAAA | - |
| *ocl4* | AGCTTGGTCATGCAGGTGTTG | CGCGCGAACTACTAGGTAAATC | - |
| *kn1* | CGGTATCTCGCTTCCATTTCAC | AGGGATCCATCTGTCAGGTTAC | - |
| *tub6* | TTCGTGGAGTGGATCCCCAACA | ACGAGGTCGTTCATGTTGCTCT | - |
| *kan1* | GATGGACGTGAAGGATCTGAC | CTGACCGTGTCCAGTGAACG | - |
| *kan2* | ACTTCCTGCAGAGCCACAC | GATCCTTCACGTCCATTAGCTC | - |
| *rs1* | AGGACAAGGAGCTCAAGTACCA | GTTGTTGATCTGCTTCTGGTCTAGG | - |
| *rld1* | GAAGCTGCCACACTTGCTAGAT | CGTAGCCCTGTTCCATTAGTTT | - |

**Regulation of Small RNA Accumulation in the Maize Shoot Apex**

F. Nogueira, D. Chitwood *et al*.

**Supplemental Table 1.** Sequences of primers used in this study.
